# Associations between cardiorespiratory fitness in youth and the incidence of site-specific cancer in men: a cohort study with register linkage

**DOI:** 10.1136/bjsports-2022-106617

**Published:** 2023-08-15

**Authors:** Aron Onerup, Kirsten Mehlig, Agnes af Geijerstam, Elin Ekblom-Bak, Hans Georg Kuhn, Lauren Lissner, Maria Åberg, Mats Börjesson

**Affiliations:** 1 Department of Pediatrics, Institute of Clinical Sciences, University of Gothenburg, Gothenburg, Sweden; 2 School of Public Health and Community Medicine, Institute of Medicine, University of Gothenburg, Gothenburg, Sweden; 3 Department of Physical Activity and Health, Swedish School of Sport and Health Sciences, GIH, Stockholm, Sweden; 4 Department of Clinical Neuroscience, Institute of Neuroscience and Physiology, Sahlgrenska Academy, University of Gothenburg, Gothenburg, Sweden; 5 Department of Molecular and Clinical Medicine, Center for Health and Performance, University of Gothenburg, Gothenburg, Sweden

**Keywords:** Sports medicine, Medical Oncology, Public health, Epidemiology

## Abstract

**Objectives:**

To assess the associations between cardiorespiratory fitness (CRF) in young men and the incidence of site-specific cancer.

**Methods:**

A Swedish population-based cohort study with register linkage of men who underwent military conscription in 1968–2005 was undertaken. CRF was assessed by maximal aerobic workload cycle test at conscription. Cox regression models assessed linear associations and included CRF, age, year and site of conscription, body mass index and parental level of education. CRF was also categorised into low, moderate and high for facilitated interpretation and results comparing high and low CRF are reported.

**Results:**

Primary analyses were performed in 1 078 000 men, of whom 84 117 subsequently developed cancer in at least one site during a mean follow-up of 33 years. Higher CRF was linearly associated with a lower hazard ratio (HR) of developing cancer in the head and neck (n=2738, HR 0.81, 95% CI 0.74 to 0.90), oesophagus (n=689, HR 0.61, 95% CI 0.50 to 0.74), stomach (n=902, HR 0.79, 95% CI 0.67 to 0.94), pancreas (n=1280, HR 0.88, 95% CI 0.76 to 1.01), liver (n=1111, HR 0.60, 95% CI 0.51 to 0.71), colon (n=3222, HR 0.82, 95% CI 0.75 to 0.90), rectum (n=2337, HR 0.95, 95% CI 0.85 to 1.05), kidney (n=1753, HR 0.80, 95% CI 0.70 to 0.90) and lung (n=1635, HR 0.58, 95% CI 0.51 to 0.66). However, higher CRF predicted a higher hazard of being diagnosed with prostate cancer (n=14 232, HR 1.07, 95% CI 1.03 to 1.12) and malignant skin cancer (n=23 064, HR 1.31, 95% CI 1.27 to 1.36).

**Conclusion:**

We report a number of protective associations between higher CRF in healthy young men and the subsequent hazard of site-specific cancers. These results have implications for public health policymaking, strengthening the incentive to promote health through improving CRF in youth.

**What is already known on this topic**

- Cardiorespiratory fitness is known to be associated with risk reductions in the development of certain site-specific cancers, but few large-scale studies of multiple cancer sites have been reported.

**What this study adds**

- Our study suggests that cardiorespiratory fitness is linearly associated with a lower hazard of developing most of the site-specific cancers assessed here, some of which have not previously been reported in relation to cardiorespiratory fitness or physical activity.

**How this study might affect research, practice or policy**

- These results strengthen the incentive for promoting interventions aimed at increasing cardiorespiratory fitness in youth.

## Introduction

While physical activity (PA) is an established risk factor for several site-specific cancers,^1^ there are fewer studies on associations between cardiorespiratory fitness (CRF) and cancer. A systematic review reported a lower risk of lung cancer (risk ratio (RR) 0.53), colorectal cancer (RR 0.74) and any cancer (RR 0.86) in men with high CRF.^2^ A slightly higher risk of being diagnosed with prostate cancer has been reported for those with high CRF (incidence rate ratio 1.10).^3^ For overall cancer, one study reported a lower risk of being diagnosed (HR 0.93 per SD increase) and of cancer-associated mortality (HR 0.82).^4^

There is a paucity of studies with a sufficiently large sample size and sufficiently long follow-up to assess the associations between CRF and the development of site-specific cancers. This can be done within the Swedish population-based registers that can be cross-linked using the unique Swedish identification number. One of the previous studies using these registers found that higher CRF was associated with increased prostate cancer diagnosis, although not with mortality or aggressive cases of prostate cancer.^3^ In another previous study, only total cancer incidence was investigated.^4^ Given the lower overall cancer risk associated with CRF and considering that prostate is the most commonly diagnosed cancer site in men, it is plausible that protective associations for other site-specific cancers, many not yet studied, can be detected in large population-based samples.

The aim of this study was to assess the associations between CRF in a large cohort of young men and the subsequent incidence of site-specific cancers.

## Methods

### Design

This is a Swedish nationwide register-based observational cohort study with prospective data. Since all data were retrieved from registers, no consent was obtained from individuals included in the study. The study is reported in accordance with the STROBE criteria and CHAMP statement.^5 6^

### Participants

All men who underwent the conscription examination in 1968–2005 at age 16–25 years, with valid information on CRF and body mass index (BMI) from conscription, were included. Exclusion criteria were a cancer diagnosis before or within 5 years after the military conscription and death or emigration within 5 years after conscription. Since CRF testing was limited to men without disease or injury and with normal electrocardiography,^7^ the study sample included men without underlying disease.

### Data sources

Conscripts were identified in the Swedish military service conscription register. Until 2010, conscription was compulsory by law for all male citizens, except for imprisoned individuals or those with severe chronic conditions or functional disabilities (2–3% annually).^7^ All conscripts underwent a standardised protocol including measurements of anthropometric measures, blood pressure, muscular strength and CRF.^8^ There were questionnaires including previous diagnoses, which included questions on smoking habits 1968–1970.

Data from conscription were linked on the person level with sociodemographic data from Statistics Sweden (∼80% coverage), the Swedish National Patient Register^9^ and the Swedish Cause of Death Register.^10^ The full dataset included information until 31 December 2019. Data cleaning was performed by KM who had access to the full dataset used to create the study dataset. All variables were described in detail in the prespecified statistical analysis plan online supplemental material.

10.1136/bjsports-2022-106617.supp1 Supplementary data

### Exposure

Information on CRF at conscription was assessed as maximal aerobic workload on a cycle ergometer test and expressed as Watt max (W_max_).^7^ After 5 min of warming up at a low resistance determined by weight, resistance was increased by 25 W per minute until interrupted by exhaustion. Bicycling was performed at 60–70 revolutions per minute, and the test officiator could choose to re-test an individual not attaining 180 heartbeats per minute.^7^ Around 1984 a change in the examination occurred, likely including a more frequent stepwise load.^7^ The results were transformed during conscription to a standardised ‘stanine’ score (1–9).^7^ Secondary analyses in our study used CRF categorised into low (1–5), moderate (6–7) and high CRF (8–9), since this categorisation has previously been shown to yield groups of similar size.^11^

Raw data of W_max_ were available for 1972–2005. In 1968–1971, raw data were not recorded into data files, but the values of W_max_ were directly converted into the standardised score. During 2000–2005, no raw data were recorded for conscripts performing the lowest three levels of fitness. We calculated W_max_/kg. For supplementary analyses, we also estimated VO_2max_ from W_max_ and body weight with a validated formula and transformed these into metabolic equivalent of task by dividing VO_2max_ by 3.5.^12^ We also categorised W_max_/kg and VO_2max_ into tertiles, stratified by conscription year to account for the change in methods over time.

### Outcome

Information on a cancer diagnosis was collected from the Swedish National Patient Register and the Cause of Death Register. Eighteen types of site-specific cancers were defined according to ICD8/9/10 codes (see online supplemental table 1). Diagnosis date refers to the first time a cancer diagnosis was registered during an inpatient or outpatient visit. With some exceptions, diagnoses of different subtypes were treated independently. For tumours in the lungs, central nervous system and liver, only diagnoses without other cancer diagnoses in the preceding years were registered to reduce the risk of misclassification of metastases.

### Covariates

#### Body mass index

Height and weight were measured and BMI was calculated as kg/m^2^. It was also categorised into underweight (<18.5 kg/m^2^), normal weight (18.5–24.9 kg/m^2^), overweight (25–29.9 kg/m^2^) and obesity (≥30 kg/m^2^).

#### Muscle strength

Two test procedures were used for muscle strength, previously described in detail.^7^ In short, isometric muscle strength was measured by knee extension (weighted 1.3), elbow flexion (weighted 0.8) and hand grip (tested with a tensiometer; weighted 1.7).

#### Parental level of education

Parental level of education was collected from Statistics Sweden and categorised according to the highest level attained by either parent: up to 9 years of compulsory school, high school to ≤2 years at university or ≥3 years at university.

#### Cognitive ability

Cognitive testing was measured in different ways over the years, although a low score was never a criterion for avoiding conscription. The test consisted of four domains, initially including verbal, spatial, logic inductive and technical ability.^7^ The four scores were converted to a 9-point stanine scale by the conscription authorities.

#### Smoking habits

In 1968–1970 and 2002–2005, questions on smoking were included in the conscription. Five categories were transformed to three categories in our analyses: no active smoking, 1–10 cigarettes or equivalent per day and >10 cigarettes or equivalent per day.

#### Patient and public involvement

Since this is a population-based study, no patient groups were involved in the planning or interpretation of the results. We plan to communicate the results to the public through mainstream media once the results are published.

#### Equity, diversity and inclusion statement

Our study included the complete male population that underwent CRF testing during military conscription during the study period. Hence, the cohort is representative of the healthy male population in Sweden. This includes men regardless of sexual or gender identity but may reduce inclusion of individuals with disabilities and individuals from other countries than Sweden, in addition to not including any women. The author team included five women and three men from different disciplines (exercise science, clinical paediatrics and oncology, biostatistics, medicine and epidemiology), including two junior scholars. We assessed the confounding by socioeconomic factors but could not assess the ethnic inequities since information on this is not available in Swedish registers.

### Statistical methods

A statistical analysis plan was specified before any statistical analyses were performed. Since this study was performed in a pre-existing cohort, no sample size calculation was performed.^13^ Being diagnosed with a cancer, death or emigration within 5 years after conscription was added as an exclusion criterion at the analysis stage to reduce the risk for reverse causality due to undiagnosed cancer.

**Figure 1:**
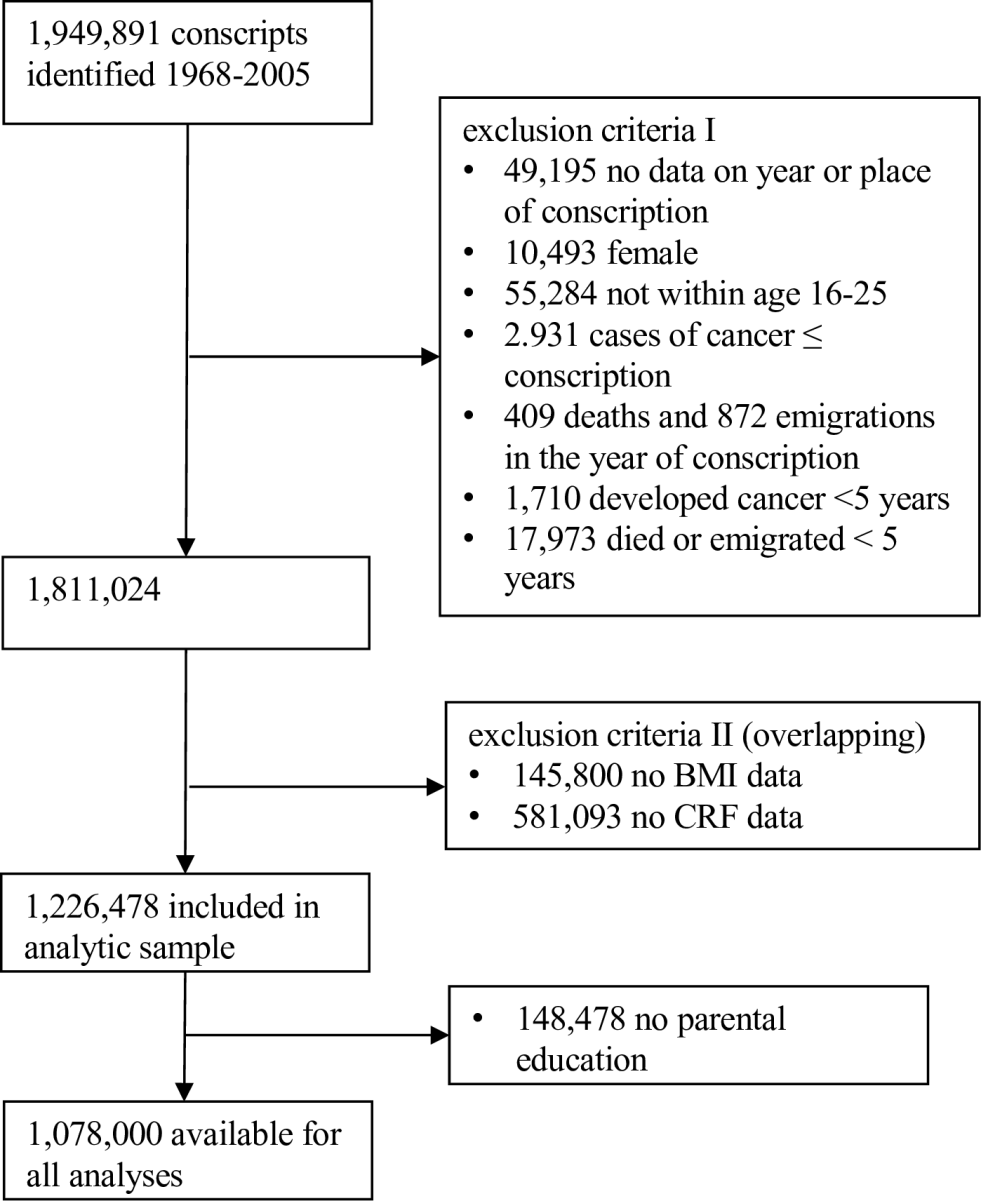
Flow chart of individuals included in the study and the main analyses. BMI, body mass index; CRF, cardiorespiratory fitness.

Cox proportional hazards models were used to explore the aim.^14^ Linearity was assessed for quantitative predictors. The proportional hazards assumption was tested by visual inspection of log-minus-log survival function plots, and assessing whether these were parallel for the three fitness categories and the various cancer outcomes. Follow-up started at year of conscription until cancer diagnosis and was censored at date of death, first emigration after conscription or end of follow-up. The main analyses assessed linear associations between the standardised CRF score (1–9) and site-specific cancer. Analyses of categorised CRF (low, moderate, high) were assessed for interpretation of the estimates. Results were given as hazard ratios (HR) with 95% CI. We created a directed acyclic graph based on existing evidence on risk factors for cancer, with a focus on identifying risk factors that are common across several cancer sites and that might be associated with CRF. The main analysis included the following covariates: year, age, site and BMI (categorised in analyses of categorised CRF, continuous in testing of linear associations) at conscription and parental education level. Missing values led to listwise deletion. Since there were no missing variables for age, year, site, CRF or BMI at conscription in the study sample, listwise deletion only occurred due to missing information on parental education (figure 1). We explored differences between the study sample and the analytical sample at baseline and performed sensitivity analyses where we assessed the associations between CRF and site-specific cancer in the full study population (with no listwise deletion). We repeated the analysis with W_max_/kg and estimated VO_2max_ as exposures. All significance tests were two-sided with a 5% significance level and performed in Stata/SE software (V.17.0).

Sensitivity analyses were performed to assess how smoking could confound the results. Associations were assessed between CRF (dichotomised) and site-specific cancer in the subpopulation where smoking information was available (n=21 480), with and without smoking as covariate, to see how including smoking changed the estimates. We also tested models including interaction terms between CRF and BMI categories with likelihood ratio testing of the estimates. Ad hoc sensitivity analyses were performed to see how adding muscle strength and cognitive ability as covariates changed the estimates. To assess the bias from listwise deletion due to missing information, we compared baseline variables for the full target population and the analytical sample.

## Results

After exclusions, 1 226 478 individuals were included in the study population (figure 1) and 1 078 000 were available for the main analyses. Mean follow-up time was 33 years. Individuals in the three fitness categories were similar in terms of age at conscription and blood pressure (table 1). Individuals with low fitness were slightly more likely to be obese than individuals with higher fitness, had a higher frequency of alcohol and substance abuse, and lower parental education than conscripts with higher CRF. Any cancer was detected in 84 117 (6.9%) of the men (see online supplemental table 2).

**Table 1:** Baseline demographics at conscription by CRF level

	Low (n=365 874)	Moderate (n=519 652)	High (n=340 952)	Overall (n=1 226 478)
Year of conscription, mean	1982	1986	1983	1984
Age at conscription, years, mean (SD)	18.4 (0.8)	18.3 (0.6)	18.3 (0.6)	18.3 (0.7)
Years of follow-up, mean (SD)	34.8 (9.7)	31.1 (10.1)	33.2 (10.6)	32.8 (10.3)
Height, cm, mean (SD)	178 (7)	179 (6)	181 (6)	179 (7)
Body mass index, kg/m^2^, mean (SD)	21.7 (3.5)	21.6 (2.5)	22.1 (2.2)	21.7 (2.8)
Body mass index category				
Underweight	49 134 (13%)	36 717 (7%)	6416 (2%)	92 267 (8%)
Normal weight	260 070 (71%)	435 774 (84%)	306 377 (90%)	1 002 221 (82%)
Overweight	44 332 (12%)	43 086 (8%)	25 835 (8%)	113 253 (9%)
Obese	12 338 (3%)	4075 (1%)	2324 (1%)	18 737 (2%)
Muscle strength category				
Low	59 363 (17%)	47 472 (10%)	13 254 (4%)	120 089 (10%)
Moderate	212 644 (61%)	289 731 (60%)	171 120 (53%)	673 495 (58%)
High	75 694 (22%)	144 881 (30%)	137 358 (43%)	357 933 (31%)
Missing	18 173 (5%)	37 568 (7%)	19 220 (6%)	74 961 (6%)
Watt_max_/kg, mean (SD)	3.40 (0.44)	4.14 (0.53)	4.63 (0.68)	4.07 (0.72)
Missing, n (%)	83 047 (23%)	73 019 (14%)	58 117 (17%)	214 183 (17%)
Estimated VO_2max_, mL O_2_*kg^–1^*min^–1^, mean (SD)	39.7 (4.7)	47.6 (5.7)	52.8 (7.2)	46.9 (7.7)
Missing, n (%)	83 047 (23%)	73 019 (14%)	58 117 (17%)	214 183 (17%)
Systolic blood pressure, mean (SD)	128 (11)	128 (11)	129 (11)	128 (11)
Missing, n (%)	495 (0.1%)	1727 (0.3%)	1217 (0.4%)	3439 (0.3%)
Diastolic blood pressure, mean (SD)	68 (10)	67 (10)	67 (10)	67 (10)
Missing, n (%)	748 (0.2%)	2148 (0.4%)	1518 (0.4%)	4414 (0.4%)
Diabetes mellitus	185 (0.05%)	180 (0.03%)	88 (0.03%)	453 (0.04%)
Hypertension	595 (0.16%)	534 (0.10%)	457 (0.13%)	1586 (0.13%)
Cardiovascular disease	7113 (1.9%)	10 095 (1.9%)	6693 (2.0%)	23 901 (2.0%)
Kidney disease	411 (0.11%)	410 (0.08%)	241 (0.07%)	1062 (0.09%)
Alcohol abuse	1434 (0.39%)	629 (0.12%)	237 (0.07%)	2300 (0.19%)
Substance abuse	2147 (0.59%)	928 (0.18%)	254 (0.07%)	3329 (0.27%)
Cognitive ability, mean (SD)	4.8 (1.9)	5.3 (1.9)	5.7 (1.8)	5.2 (1.9)
Missing, n (%)	57 304 (16%)	51 345 (10%)	40 787 (12%)	149 436 (12%)
Parental education				
Compulsory school	110 488 (36%)	115 611 (25%)	73 089 (24%)	299 188 (28%)
High school ≤2 years university	166 813 (54%)	272 474 (58%)	163 174 (54%)	602 461 (56%)
>2 years university	31 673 (10%)	80 592 (17%)	64 086 (21%)	176 351 (16%)
Missing	56 900 (16%)	50 975 (10%)	40 603 (12%)	148 478 (12%)
Smoking 1968–1970	8872	6539	5896	21 307
No active smoking	2779 (31%)	2556 (39%)	3197 (54%)	8532 (40%)
Smoking 1–10 cigarettes	3039 (34%)	2164 (33%)	1658 (28%)	6861 (32%)
Smoking >10 cigarettes	2865 (32%)	1705 (26%)	908 (15%)	5478 (26%)
Missing	189 (2%)	114 (2%)	133 (2%)	436 (2%)
Smoking 2002–2005	8973	30 699	14 486	54 158
No active smoking	7691 (86%)	28 579 (93%)	14 221 (98%)	50 491 (93%)
Smoking 1–10 cigarettes	1024 (11%)	1797 (6%)	237 (2%)	3058 (6%)
Smoking >10 cigarettes	258 (3%)	323 (1%)	28 (0%)	609 (1%)

For those who conscripted in 1968–1970 (n=21 307), 60% reported any current smoking and men with higher CRF reported lower proportions of active smoking: low 67%, moderate 59% and high 44% (table 1). In 2002–2005, only 7% of conscripts reported any current smoking.

### Malignant skin cancer

**Figure 2:**
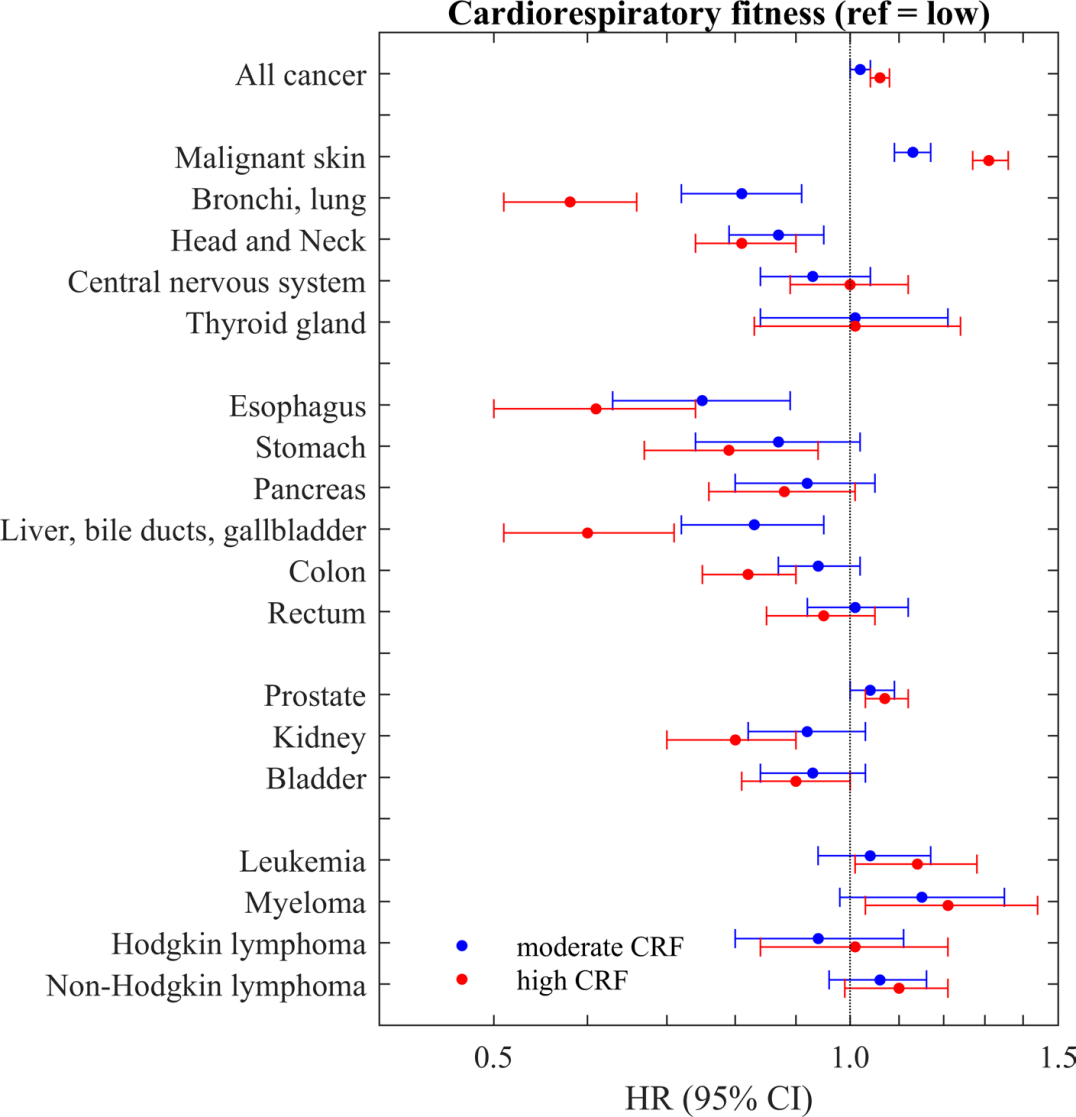
Forest plot showing the results for CRF at conscription and incidence of site-specific cancer (numbers in table 2). CRF, cardiorespiratory fitness.

The hazard of developing malignant skin cancer was linearly associated with CRF, with those having high CRF having the highest risk (table 2 and figure 2). The sensitivity analysis for smoking did not change the estimates (table 3).

**Table 2:** CRF at conscription and incidence of cancer (n=1 078 000)

Cancer site	No of cases	Cardiorespiratory fitness (ref=low)		
		Moderate	High	P value for linear trend^*^
		HR (95% CI)	HR (95% CI)	
Any cancer site	64 609	1.02 (1.00 to 1.04)	1.06 (1.04 to 1.08)	<0.001
Malignant skin	23 064	1.13 (1.09 to 1.17)	1.31 (1.27 to 1.36)	<0.001
Bronchi and lung	1635	0.81 (0.72 to 0.91)	0.58 (0.51 to 0.66)	<0.001
Head and neck	2738	0.87 (0.79 to 0.95)	0.81 (0.74 to 0.90)	<0.001
CNS	2014	0.93 (0.84 to 1.04)	1.00 (0.89 to 1.12)	0.91
Thyroid gland	685	1.01 (0.84 to 1.21)	1.01 (0.83 to 1.24)	0.89
Gastrointestinal cancers				
Oesophagus	689	0.75 (0.63 to 0.89)	0.61 (0.50 to 0.74)	<0.001
Stomach	902	0.87 (0.74 to 1.02)	0.79 (0.67 to 0.94)	<0.001
Pancreas	1280	0.92 (0.80 to 1.05)	0.88 (0.76 to 1.01)	<0.001
Liver, bile ducts and gallbladder	1111	0.83 (0.72 to 0.95)	0.60 (0.51 to 0.71)	<0.001
Colon	3222	0.94 (0.87 to 1.02)	0.82 (0.75 to 0.90)	<0.001
Rectum	2337	1.01 (0.92 to 1.12)	0.95 (0.85 to 1.05)	0.03
Urological cancers				
Prostate	14 232	1.04 (1.00 to 1.09)	1.07 (1.03 to 1.12)	<0.001
Kidney	1753	0.92 (0.82 to 1.03)	0.80 (0.70 to 0.90)	<0.001
Bladder	2259	0.93 (0.84 to 1.03)	0.90 (0.81 to 1.00)	0.02
Haematological cancers				
Leukaemia	1991	1.04 (0.94 to 1.17)	1.14 (1.01 to 1.28)	0.20
Myeloma	915	1.15 (0.98 to 1.35)	1.21 (1.03 to 1.44)	0.02
Hodgkin’s lymphoma	843	0.94 (0.80 to 1.11)	1.01 (0.84 to 1.21)	0.61
Non-Hodgkin’s lymphoma	2559	1.06 (0.96 to 1.16)	1.10 (0.99 to 1.21)	0.06

Adjusted for year, site, age (categorical CRF for BMI categories, linear trend for continuous BMI) and parental education at conscription.

*Linear trends tested using 9-level CRF.

BMI, body mass index; CNS, central nervous system; CRF, cardiorespiratory fitness.

**Table 3:** CRF and cigarette smoking at conscription and future risk of cancer in individuals who underwent conscription in 1968–1970 when information on smoking habits was collected (n=21 307)

Cancer site	No of cases	CRF (ref=low)	Smoking (ref=no smoking)	
		Moderate/high	1–10 cigarettes/day	>10 cigarettes/day
		HR (95% CI)	HR (95% CI)	HR (95% CI)
Any cancer	4371	1.05 (0.99 to 1.11)	NA	NA
**Adjusted for smoking**		1.06 (1.00 to 1.13)	1.01 (0.94 to 1.08)	1.11 (1.02 to 1.19)
Malignant skin	1223	1.22 (1.08 to 1.37)	NA	NA
**Adjusted for smoking**		1.17 (1.03 to 1.31)	0.80 (0.70 to 0.91)	0.70 (0.60 to 0.81)
Bronchi and lung	245	0.75 (0.58 to 0.97)	NA	NA
**Adjusted for smoking**		0.92 (0.71 to 1.18)	2.50 (1.67 to 3.74)	5.68 (3.90 to 8.26)
Head and neck	193	0.72 (0.54 to 0.96)	NA	NA
**Adjusted for smoking**		0.78 (0.59 to 1.04)	1.53 (1.07 to 2.20)	2.00 (1.39 to 2.89)
Gastrointestinal cancers				
Oesophagus	83	0.65 (0.42 to 1.01)	NA	NA
**Adjusted for smoking**		0.76 (0.49 to 1.18)	2.62 (1.41 to 4.87)	3.82 (2.07 to 7.06)
Stomach	71	0.71 (0.44 to 1.13)	NA	NA
**Adjusted for smoking**		0.77 (0.48 to 1.24)	2.11 (1.17 to 3.81)	1.94 (1.03 to 3.67)
Pancreas	119	0.81 (0.56 to 1.17)	NA	NA
**Adjusted for smoking**		0.86 (0.60 to 1.25)	1.44 (0.91 to 2.26)	1.65 (1.03 to 2.62)
Liver, bile ducts and gallbladder	97	0.79 (0.53 to 1.18)	NA	NA
**Adjusted for smoking**		0.87 (0.58 to 1.30)	1.75 (1.03 to 2.95)	2.12 (1.25 to 3.59)
Colon	213	0.98 (0.74 to 1.29)	NA	NA
**Adjusted for smoking**		0.98 (0.74 to 1.29)	1.05 (0.77 to 1.44)	0.96 (0.67 to 1.37)
Rectum	156	1.40 (1.00 to 1.95)	NA	NA
**Adjusted for smoking**		1.42 (1.02 to 2.00)	1.15 (0.80 to 1.67)	1.16 (0.77 to 1.74)
Urological cancers				
Prostate	1520	1.10 (0.99 to 1.22)	NA	NA
**Adjusted for smoking**		1.09 (0.98 to 1.21)	0.92 (0.82 to 1.03)	0.92 (0.81 to 1.05)
Kidney	115	1.09 (0.75 to 1.60)	NA	NA
**Adjusted for smoking**		1.14 (0.78 to 1.68)	0.97 (0.61 to 1.54)	1.48 (0.95 to 2.32)
Bladder	217	1.16 (0.88 to 1.53)	NA	NA
**Adjusted for smoking**		1.29 (0.98 to 1.71)	1.81 (1.28 to 2.55)	2.45 (1.73 to 3.47)
Non-Hodgkin’s lymphoma	133	1.09 (0.77 to 1.55)	NA	NA
**Adjusted for smoking**		1.16 (0.81 to 1.66)	1.59 (1.05 to 2.40)	1.62 (1.03 to 2.52)

Adjusted for conscription year, age, and site and BMI at conscription. Where indicated also adjusted for smoking. Models did not converge for central nervous system, thyroid gland, leukaemia, myeloma, and Hodgkin’s lymphoma due to few events.

CRF, cardiorespiratory fitness.

### Bronchi and lungs

There were dose-response associations between lower CRF and the hazard of developing lung cancer (table 2). However, the association with CRF was mainly explained by smoking status (table 3).

### Head and neck

The hazard of developing head and neck cancer was linearly associated with lower CRF (table 2). Smoking was a risk factor for head and neck cancer, but hardly changed the estimates (table 3).

### Central nervous system

The hazard of developing tumours in the central nervous system was not associated with CRF (table 2).

### Thyroid gland

We could not detect any association between CRF and cancer in the thyroid gland (table 2).

### Gastrointestinal cancer

There were linear associations between lower CRF and the hazard of developing cancer in the oesophagus, stomach, pancreas, liver, colon and the rectum (table 2). The estimates for the associations between CRF and cancer changed marginally for most of the gastrointestinal cancers when adjusting for smoking. However, for cancers in the oesophagus and liver, approximately one-third of the hazard reductions were removed after adjusting for smoking (table 3).

### Urological system

There was a linear association between CRF and the hazard of developing prostate cancer, with the highest hazard for those with high CRF (table 2). Adding smoking as covariate did not change the estimates (table 3).

There were linear associations between low CRF and the hazard of developing kidney cancer (table 2). The estimates did not change considerably when adjusting for smoking (table 3). There was a weak association between CRF and the hazard of developing bladder cancer (table 2), which seemed to be at least partially confounded by smoking (table 3).

### Haematological malignancies

There were no associations between CRF and the hazard of developing leukaemia or Hodgkin’s or non-Hodgkin’s lymphomas, but a weak association between a higher CRF and a higher hazard of developing myeloma (table 2), which was not seen in the supplementary analyses of W_max_/kg (table 4).

**Table 4:** W_max_/kg and estimated VO_2max_ at conscription and incidence of cancer (n=1 012 295)

Cancer site	No of cases	Middle tertile^†^	Highest tertile^†^	Analysis of continuous W_max_/kg^‡^		Analysis of continuous VO_2max_^§^		Change per MET
		HR (95% CI)	HR (95% CI)	P value	HR (95% CI)	P value	HR (95% CI)	HR (95% CI)
Any cancer site	53 841	0.99 (0.97 to 1.01)	0.98 (0.96 to 1.01)	0.39	0.99 (0.98 to 1.01)	0.39	1.00 (1.00 to 1.00)	1.00 (0.99 to 1.00)
Malignant skin	19 741	1.08 (1.04 to 1.12)	1.19 (1.14 to 1.23)	<0.001	1.14 (1.12 to 1.17)	<0.001	1.01 (1.01 to 1.01)	1.05 (1.04 to 1.05)
Bronchi and lung	1207	0.89 (0.78 to 1.02)	0.62 (0.53 to 0.72)	<0.001	0.64 (0.57 to 0.71)	<0.001	0.96 (0.95 to 0.97)	0.86 (0.83 to 0.89)
Head and neck	2305	0.90 (0.81 to 0.99)	0.78 (0.70 to 0.86)	<0.001	0.85 (0.79 to 0.92)	<0.001	0.99 (0.98 to 0.99)	0.95 (0.93 to 0.97)
CNS	1822	0.97 (0.86 to 1.08)	0.92 (0.82 to 1.04)	0.03	0.92 (0.85 to 0.99)	0.03	0.99 (0.98 to 1.00)	0.97 (0.95 to 1.00)
Thyroid gland	610	0.91 (0.75 to 1.11)	0.78 (0.63 to 0.96)	0.14	0.90 (0.79 to 1.03)	0.14	0.99 (0.98 to 1.00)	0.97 (0.93 to 1.01)
Gastrointestinal cancers								
Oesophagus	537	0.68 (0.55 to 0.85)	0.60 (0.48 to 0.75)	<0.001	0.64 (0.54 to 0.76)	<0.001	0.96 (0.94 to 0.97)	0.87 (0.82 to 0.91)
Stomach	726	0.79 (0.66 to 0.95)	0.71 (0.59 to 0.87)	<0.001	0.71 (0.62 to 0.81)	<0.001	0.97 (0.96 to 0.98)	0.89 (0.85 to 0.93)
Pancreas	1022	0.93 (0.80 to 1.08)	0.71 (0.61 to 0.84)	<0.001	0.76 (0.67 to 0.85)	<0.001	0.97 (0.96 to 0.99)	0.91 (0.88 to 0.95)
Liver, bile ducts and gallbladder	917	0.85 (0.72 to 0.99)	0.64 (0.54 to 0.76)	<0.001	0.69 (0.61 to 0.78)	<0.001	0.97 (0.95 to 0.98)	0.89 (0.85 to 0.92)
Colon	2663	0.88 (0.80 to 0.97)	0.79 (0.71 to 0.87)	<0.001	0.85 (0.80 to 0.92)	<0.001	0.99 (0.98 to 0.99)	0.95 (0.93 to 0.97)
Rectum	1922	0.99 (0.88 to 1.10)	0.85 (0.76 to 0.96)	0.001	0.86 (0.80 to 0.94)	0.001	0.99 (0.98 to 0.99)	0.95 (0.93 to 0.98)
Urological cancers								
Prostate	10 504	1.06 (1.01 to 1.12)	1.05 (0.99 to 1.10)	0.008	1.05 (1.01 to 1.09)	0.008	1.00 (1.00 to 1.01)	1.02 (1.00 to 1.03)
Kidney	1452	0.81 (0.71 to 0.92)	0.63 (0.55 to 0.72)	<0.001	0.73 (0.67 to 0.81)	<0.001	0.97 (0.96 to 0.98)	0.90 (0.87 to 0.93)
Bladder	1748	1.04 (0.92 to 1.16)	0.90 (0.79 to 1.01)	0.003	0.88 (0.80 to 0.96)	0.003	0.99 (0.98 to 1.00)	0.96 (0.93 to 0.99)
Haematological cancers								
Leukaemia	1721	0.86 (0.76 to 0.97)	0.95 (0.84 to 1.07)	0.20	0.95 (0.87 to 1.03)	0.20	0.99 (0.99 to 1.00)	0.98 (0.96 to 1.01)
Myeloma	749	0.96 (0.80 to 1.15)	0.99 (0.83 to 1.20)	0.89	0.99 (0.87 to 1.13)	0.89	1.00 (0.99 to 1.01)	1.00 (0.96 to 1.04)
Hodgkin’s lymphoma	782	1.05 (0.88 to 1.25)	0.94 (0.78 to 1.14)	0.49	0.96 (0.85 to 1.08)	0.49	1.00 (1.01 to 1.06)	0.99 (0.95 to 1.02)
Non-Hodgkin’s lymphoma	2247	0.90 (0.81 to 1.00)	0.93 (0.83 to 1.03)	0.33	0.97 (0.90 to 1.04)	0.33	1.00 (0.99 to 1.00)	0.99 (0.97 to 1.01)

Adjusted for year, site, age, body mass index and parental education at conscription.

Reference=lowest tertile.

*Estimated from W_max_ using the formula by Kokkinos *et al*.^12^

†Tertiles of W_max_/kg and estimated VO_2max_, stratified by conscription year (same results for both variables).

‡Linear trends and estimate per W/kg for continuous W_max_/kg.

§Linear trends and estimate per mL O_2_*kg^–1^*min^–1^ for continuous VO_2max_.

CNS, central nervous system; MET, Metabolic equivalent of task (3.5 mL O_2_*kg^–1^*min^–1^).

### Any cancer

There were linear associations between CRF and the hazard of developing any cancer (table 2). However, the estimates were close to 1, and we did not find any association in the supplementary analyses using W_max_/kg (table 4).

### Supplementary analyses

The analyses using W_max_/kg and estimated VO_2max_ showed similar results to the primary analyses, with 1–3% lower risk per mL O_2_*kg^–1^*min^–1^ increase for the site-specific cancers with linear associations (table 4). However, the higher hazard of myeloma and any cancer with higher CRF seen in the primary analyses were not seen when using VO_2max_. The results for tertiles of W_max_/kg and estimated VO_2max_ were the same, since VO_2max_ was derived from W_max_ and weight. Table 4 also shows the HR per metabolic equivalent of task.

The interaction analysis for interactions between CRF and BMI on the associations with site-specific cancers revealed no interactions for most of the sites (online supplemental table 4). However, for bladder cancer, moderate/high CRF was not associated with any change in hazard compared with low CRF in participants with normal weight, but with a considerably lower hazard in overweight/obese participants. For non-Hodgkin’s lymphoma, moderate/high CRF was associated with a tendency towards lower hazard compared with low CRF for individuals with overweight/obesity but with a higher hazard in normal weight participants.

Adding muscle strength as a covariate had little effect on the associations between CRF and cancer, except for the association with rectal cancer which was marginally weakened and no longer statistically significant (see online supplemental table 3). Adjusting for cognitive ability had little effect on the estimates, while the associations between CRF and rectal and bladder cancers were no longer significant, where statistical significance for the association between CRF and non-Hodgkin’s lymphoma changed from 0.06 to 0.02 (see online supplemental table 5). The dropout analysis showed that baseline variables were distributed similarly in the target population (n=1 830 707) and the analysis sample, except for diabetes which was 10 times more common in the full population (see online supplemental table 6). Since this difference was due to conscripts with underlying disease, injury or pathological resting ECG being excluded from CRF testing, we performed a sensitivity analysis excluding all participants with chronic disease at conscription. This resulted in similar results to the main analysis (see online supplemental table 7). Online supplemental table 6 also shows that the study sample and the population with data on all variables included in the main analysis, including parental education, were almost identical at baseline (online supplemental table 6). The sensitivity analysis in the full study sample, without adjusting for parental education, did not change the interpretation for any cancer site and the HRs were similar to those in the primary analyses (see online supplemental table 8).

## Discussion

This large population-based study of Swedish men presents novel results on the associations between CRF in youth and 18 site-specific cancers in men. To our knowledge, we show for the first time that higher CRF is associated with a lower hazard for cancer in the head and neck, oesophagus, stomach, pancreas, liver, colon, rectum and kidney. The results indicate a 20–40% lower hazard for men with high versus low CRF for several gastrointestinal sites, which would be clinically relevant.

Previous studies have looked at the association between CRF and the risk of developing colorectal cancer.^2^ Our study showed that CRF was associated with a reduced hazard of colon cancer, with a hazard reduction of 21% for high CRF. However, the association was weaker for rectal cancer. The novel reports of associations between CRF and cancer in the oesophagus, stomach, colon, liver and kidney in our study are supported by similar associations previously reported for PA.^1^ There are site-specific cancers for which previous studies have been unable to conclude whether there is an association for either CRF or PA. We can report dose-dependent associations between CRF and cancer in the head and neck and pancreas. For lung cancer, a systematic review reported a 50% reduced risk for high versus low CRF,^2^ while the WHO concluded that the associations for PA might be confounded by smoking.^1^ Our results confirmed a hazard reduction which seemed to be confounded by smoking.

For prostate cancer and malignant skin cancer, higher CRF was associated with a higher hazard. For prostate cancer, this is in line with previous studies, one of which was performed on the same registers.^3 15^ The authors of the previous study reported that the association between CRF and prostate cancer only occurred for any cancer diagnosis and did not apply to aggressive prostate cancer nor prostate cancer mortality. They concluded that this was probably explained by increased prostate cancer screening.^3^ In our study we show that the association was not confounded by smoking. For malignant skin cancer, the increase with higher CRF could possibly be due to a higher UV exposure for those with higher CRF. Our data did not allow adjustment for UV exposure.

Previous studies have shown a lower hazard across all cancer sites for individuals with higher CRF, with a HR of 0.86 in a 2019 systematic review.^2^ In our study we report a higher hazard with higher CRF. This is explained by higher hazard for the two major cancer sites, prostate and malignant skin cancer, with the probable confounding previously discussed. This confounding might explain the differences in results between different populations and time periods, with varying associations between CRF and UV exposure and cancer screening. Combined, the results for overall cancer highlight the need for analyses of site-specific cancers, since specific residual confounding may exist for specific site-specific cancers, as shown for lung cancer, malignant skin cancer and prostate cancer in the current study. The interaction analysis between CRF and BMI showed that the protective associations between CRF and site-specific cancers were generally not dependent on BMI, indicating that increasing CRF is beneficial, regardless of body weight. For bladder cancer and non-Hodgkin’s lymphoma, the preventive association with CRF was only seen in participants with overweight/obesity. This is in line with the fat but fit paradigm,^16^ indicating that maintaining a healthy CRF could be of even more importance for individuals with overweight/obesity.

This study has a number of strengths, including the population-based approach, the use of prospectively registered data with high validity, the large sample size and the long follow-up. These strengths increase both internal validity and generalisability and contribute to the novel results for several site-specific cancers. A limitation is the observational design, limiting conclusions on causality. The sensitivity analyses showed that the associations between CRF and site-specific cancers were robust to excluding pre-existing chronic disease, to adjusting for muscle strength and for cognitive ability and that participants missing information on parental education were similar to the analytical sample at baseline and had similar associations between CRF and site-specific cancer. The major limitation of this study is the lack of full data on other known lifestyle risk factors, especially smoking, which increases the risk of confounding. There is also a possibility of measurement error in smoking, with the relatively crude assessment. We have used the information on smoking habits from a subpopulation of more than 20 000 individuals for which this information was available to see how adjusting for smoking changed the estimates. This approach detected the anticipated confounding for lung cancer and partial confounding for cancers in the liver and oesophagus. Thus, we believe that this sensitivity analysis helps inform about cancer sites where confounding from smoking is probable and that our study improves the evidence compared with previous studies where information on smoking has been lacking.^3 4^ Furthermore, smoking declined dramatically in Sweden during the study period. Hence, confounding from smoking in the full population should be lower than that observed in the 1968–70 cohort where smoking was frequent. Our study is limited by a lack of information on other lifestyle risk factors such as alcohol and diet as well as other unmeasured confounding. The use of a 9-grade scale rather than widely accepted measures of CRF such as W_max_ or VO_2max_ is also a limitation. However, the CRF measure used comes from a maximal ergometer test and has been shown to predict several other health outcomes. Another limitation is the lack of data on changes in exposure during the long follow-up period, despite CRF being a time-varying exposure. While CRF decreases through life, it generally tracks stronger than PA.^17 18^ There is also a possible feedback between CRF and BMI over time. Hence, studies assessing the effect of CRF at different ages on site-specific cancers are warranted. The fact that CRF testing was only performed for men without underlying disease makes our results valid only for young men without chronic disease. Our sensitivity analysis showed that the results did not change when excluding participants, indicating that men with chronic disease could also have cancer protective effects from CRF. We did not perform any specific handling of competing events.^19^ However, since we did not estimate any cumulative incidence and the HR from Cox regression is robust to competing events,^20^ our results should be valid despite probable differences in the risk of death from, for example, cardiovascular disease between the groups. The HRs in our study do not account for possible time-dependent differences in hazard during follow-up, which is a limitation.^21^

This study has public health implications. While the CRF response to exercise (trainability) has a relatively strong genetic component,^22^ it is also correlated with the amount of PA of sufficient intensity.^23^ It is possible that part of the associations in our study could be explained by shared genetic variation, previously reported for cardiovascular disease.^24^ To our knowledge, shared genetic variations between CRF and cancer are yet to be reported. Since PA is an established risk factor for several site-specific cancers according to the WHO,^1^ we consider it reasonable to hypothesise that the associations between CRF and site-specific cancers in our study are mainly explained by a difference in underlying PA. While the reported risk reductions for PA are 10–20% for most cancers where there is an association, our study showed 20–30% hazard reductions for several site-specific cancers. One explanation for this could be that most studies on PA use self-reported doses of PA, which has a relatively low sensitivity. However, CRF is improved mainly by aerobic PA of moderate to high relative intensity and less by low intensity PA.^25^ Thus, our results may indicate that public health efforts aimed at reducing cancer should focus on aerobic PA of sufficient relative intensity to increase CRF. This has been suggested in studies on all-cause mortality,^25^ and is also reflected in the American Society of Clinical Oncology guidelines on exercise during cancer treatment,^26^ focusing on aerobic exercise training.

Our results indicate the following future research directions: (1) to confirm the results for cancer sites that have not been previously reported; (2) to clarify the effect of fitness on cancer in various periods of life; and (3) to establish whether a reduced hazard of developing cancer also translates into increased survival after being diagnosed with cancer.

## Conclusion

This study shows that higher fitness in healthy young men is associated with a lower hazard of developing 9 out of 18 investigated site-specific cancers, with the most clinically relevant hazard rates in the gastrointestinal tract. These results could be used in public health policymaking, further strengthening the incentive for promoting interventions aimed at increasing CRF in youth.

## Data Availability

No data are available. The data in this study are not available for data sharing.
