# Supplementary material for: Associations between cardiorespiratory fitness in youth and the incidence of site-specific cancer in men: a cohort study with register linkage
Source: Br J Sports Med. 2023 Aug 15;57(19):1248–56. doi: 10.1136/bjsports-2022-106617 (PMC10579181; doi:10.1136/bjsports-2022-106617)
Supplement: Supplementary data [file bjsports-2022-106617supp001.pdf]

Supplementary table 1. Definitions for all site-specific cancers according to ICD diagnosis.

| Type of cancer | ICD-8 | ICD-9 | ICD-10 |
|---|---|---|---|
| **Prostate** | 185 | 185 | C61 |
| **Bronchi and lung** | 162 | 162 | C34 |
| **Colon** | 153 | 153 | C18 |
| **Rectum** | 154 | 154 | C19 & C20 |
| **Malignant skin** | 172-173 | 172-173 | C43-C44 |
| **Non-Hodgkin's lymphoma** | 200 | 200 | C82-C86 |
| **Renal** | 189 | 189 | C64 |
| **Head-Neck** | 140-149 | 140-149 | C00-C14 C30-C32 |
| **Central nervous system** | 191-192 | 191-192, 194D & 194E | C70- C72, C75.1-C75.3 |
| **Pancreas** | 157 | 157 | C25 |
| **Bladder** | 188 | 188 | C67 |
| **Esophagus** | 150 | 150 | C15 |
| **Stomach** | 151 | 151 | C16 |
| **Liver, bile ducts and gallbladder** | 155-156 | 155-156. Not 155C | C22-C24. Not C22.9. |
| **Myeloma** | 203 | 203 | C90 |
| **Thyroid gland** | 193 | 193 | C73 |
| **Hodgkin's lymphoma** | 201 | 201 | C81 |
| **Leukemia** | 204-207 | 204-208 | C91-C95 |

Supplementary table 2. Absolute numbers for outcomes, in total and by level of fitness at conscription.

| Outcome | Low (N=368,971) | Moderate (N=523,970) | High (N=343,648) | Total (N=1,236,589) |
|---|---|---|---|---|
| | N (%) | N (%) | N (%) | N (%) |
| **Any cancer** | 28,430 (7.8 %) | 30,485 (5.9 %) | 25,202 (7.4 %) | 84,117 (6.9 %) |
| **Malignant skin** | 8,547 (2.3 %) | 10,303 (2.0 %) | 9,440 (2.8 %) | 28,290 (2.3 %) |
| **Bronchi and lung** | 1,181 (0.3 %) | 857 (0.2 %) | 529 (0.2 %) | 2,567 (0.2 %) |
| **Head & Neck** | 1,359 (0.4 %) | 1,242 (0.2 %) | 943 (0.3 %) | 3,544 (0.3 %) |
| **Central nervous system** | 942 (0.3 %) | 1,046 (0.2 %) | 852 (0.3 %) | 2,840 (0.2 %) |
| **Thyroid gland** | 262 (0.1 %) | 327 (0.1 %) | 233 (0.1 %) | 822 (0.1 %) |
| **GASTROINTESTINAL CANCERS** | | | | |
| **Esophagus** | 436 (0.1 %) | 333 (0.1 %) | 240 (0.1 %) | 1,009 (0.1 %) |
| **Stomach** | 503 (0.1 %) | 458 (0.1 %) | 331 (0.1 %) | 1,292 (0.1 %) |
| **Liver, bile ducts and gallbladder** | 703 (0.2 %) | 557 (0.1 %) | 333 (0.1 %) | 1,593 (0.1 %) |
| **Pancreas** | 693 (0.2 %) | 661 (0.1 %) | 505 (0.2 %) | 1,859 (0.2 %) |
| **Colon** | 1,610 (0.4 %) | 1,598 (0.3 %) | 1,093 (0.3 %) | 4,301 (0.4 %) |
| **Rectum** | 1,128 (0.3 %) | 1,152 (0.2 %) | 857 (0.3 %) | 3,137 (0.3 %) |
| **UROLOGICAL CANCERS** | | | | |
| **Prostate** | 6,766 (1.9 %) | 6,745 (1.3 %) | 6,168 (1.8 %) | 19,679 (1.6 %) |
| **Bladder** | 1,164 (0.3 %) | 1,071 (0.2 %) | 835 (0.2 %) | 3,070 (0.3 %) |
| **Kidney** | 874 (0.2 %) | 834 (0.2 %) | 586 (0.2 %) | 2.294 (0.2 %) |
| **HEMATOLOGICAL CANCERS** | | | | |
| **Myeloma** | 378 (0.1 %) | 441 (0.1 %) | 394 (0.1 %) | 1,213 (0.1 %) |
| **Non-Hodgkin's lymphoma** | 1,050 (0.3 %) | 1,228 (0.2 %) | 972 (0.3 %) | 3,250 (0.3 %) |
| **Hodgkin's lymphoma** | 324 (0.1 %) | 407 (0.1 %) | 307 (0.1 %) | 1,038 (0.1 %) |
| **Leukemia** | 881 (0.2 %) | 1,007 (0.2 %) | 842 (0.3 %) | 2,730 (0.2 %) |

Supplementary table 3. CRF at conscription and incidence of cancer (n=1,005,911), muscle strength included as covariate.

| | | Cardiorespiratory fitness (ref = low) | | |
|---|---|---|---|---|
| **Cancer site** | **n cases** | **Moderate** | **High** | |
| | | **HR (95% CI)** | **HR (95% CI)** | **P** |
| **Any cancer site** | 62,748 | 1.01 (0.99-1.03) | 1.04 (1.02-1.07) | <0.001 |
| **Malignant skin** | 22,405 | 1.12 (1.08-1.15) | 1.29 (1.24-1.34) | <0.001 |
| **Bronchi and lung** | 1,596 | 0.82 (0.73-0.92) | 0.58 (0.51-0.67) | <0.001 |
| **Head & Neck** | 2,666 | 0.84 (0.77-0.92) | 0.78 (0.70-0.86) | <0.001 |
| **Central nervous system** | 1,926 | 0.94 (0.84-1.05) | 1.01 (0.90-1.14) | 0.75 |
| **Thyroid gland** | 643 | 1.05 (0.86-1.27) | 1.06 (0.86-1.31) | 0.71 |
| **GASTROINTESTINAL CANCERS** | | | | |
| **Esophagus** | 680 | 0.75 (0.63-0.90) | 0.59 (0.48-0.72) | <0.001 |
| **Stomach** | 880 | 0.86 (0.73-1.01) | 0.75 (0.63-0.90) | <0.001 |
| **Pancreas** | 1,257 | 0.92 (0.81-1.06) | 0.89 (0.77-1.19) | 0.001 |
| **Liver, bile ducts and gallbladder** | 1,090 | 0.81 (0.71-0.94) | 0.59 (0.50-0.69) | <0.001 |
| **Colon** | 3,143 | 0.94 (0.87-1.03) | 0.84 (0.76-0.92) | <0.001 |
| **Rectum** | 2,281 | 1.03 (0.93-1.14) | 0.96 (0.86-1.07) | 0.09 |
| **UROLOGICAL CANCERS** | | | | |
| **Prostate** | 14,021 | 1.03 (0.98-1.07) | 1.04 (1.00-1.09) | 0.001 |
| **Kidney** | 1,724 | 0.91 (0.82-1.02) | 0.80 (0.70-0.91) | <0.001 |
| **Bladder** | 2,217 | 0.92 (0.84-1.02) | 0.89 (0.80-0.99) | 0.01 |
| **HEMATOLOGICAL CANCERS** | | | | |
| **Leukemia** | 1,910 | 1.05 (0.94-1.17) | 1.11 (0.98-1.25) | 0.46 |
| **Myeloma** | 895 | 1.15 (0.98-1.36) | 1.21 (1.01-1.44) | 0.03 |
| **Hodgkin's lymphoma** | 794 | 0.91 (0.77-1.08) | 0.99 (0.82-1.19) | 0.39 |
| **Non-Hodgkin's lymphoma** | 2,471 | 1.05 (0.95-1.16) | 1.09 (0.98-1.21) | 0.06 |

Covariates included in analysis: CRF, muscle strength, site of conscription, year of conscription, parental education, age at conscription, and BMI. CRF=cardiorespiratory fitness. HR=Hazard ratio

Supplementary table 4. Interaction analyses between CRF and BMI for the association with site-specific cancer, as well as hazard ratios for the association between CRF and site-specific cancer, stratified by BMI (N=1,078,000).

| Cancer site | Interaction with BMI | Underweight/normal weight | Overweight/obesity |
|---|---|---|---|
| | P | HR (95% CI) | HR (95% CI) |
| **Malignant skin, low CRF** | 0.33 | Ref | 0.85 (0.78-0.92) |
| *Moderate/high CRF* | | 1.2 (1.17-1.24) | 1.07 (1.01-1.14) |
| **Bronchi and lung, low CRF** | 0.08 | Ref | 0.96 (0.71-1.29) |
| *Moderate/high CRF* | | 0.67 (0.60-0.74) | 0.88 (0.72-1.08) |
| **Head & Neck, low CRF** | 0.64 | Ref | 1.19 (0.97-1.46) |
| *Moderate/high CRF* | | 0.85 (0.79-0.93) | 0.95 (0.81-1.12) |
| **Central nervous system, low CRF** | 0.73 | Ref | 1.07 (0.85-1.35) |
| *Moderate/high CRF* | | 0.98 (0.88-1.08) | 1.00 (0.82-1.21) |
| **Thyroid gland, low CRF** | 0.23 | Ref | 0.99 (0.66-1.48) |
| *Moderate/high CRF* | | 0.99 (0.83-1.18) | 1.32 (0.97-1.81) |
| **GASTROINTESTINAL CANCERS** | | | |
| **Esophagus, low CRF** | 0.86 | Ref | 2.19 (1.55-3.09) |
| *Moderate/high CRF* | | 0.71 (0.60-0.83) | 1.49 (1.14-1.95) |
| **Stomach, low CRF** | 0.46 | Ref | 2.18 (1.62-2.94) |
| *Moderate/high CRF* | | 0.86 (0.74-1.00) | 1.63 (1.28-2.07) |
| **Pancreas, low CRF** | 0.37 | Ref | 1.37 (1.01-1.88) |
| *Moderate/high CRF* | | 0.88 (0.78-1.00) | 1.43 (1.16-1.77) |
| **Liver, bile ducts and gallbladder, low CRF** | 0.25 | Ref | 1.77 (1.34-1.32) |
| *Moderate/high CRF* | | 0.76 (0.66-0.86) | 1.08 (0.85-1.37) |
| **Colon, low CRF** | 0.35 | Ref | 1.40 (1.17-1.67) |
| *Moderate/high CRF* | | 0.90 (0.83-0.97) | 1.13 (0.98-1.31) |
| **Rectum, low CRF** | 0.27 | Ref | 1.35 (1.07-1.69) |
| *Moderate/high CRF* | | 0.99 (0.91-1.09) | 1.14 (0.96-1.36) |
| **UROLOGICAL CANCERS** | | | |
| **Prostate, low CRF** | 0.74 | Ref | 0.88 (0.77-1.01) |
| *Moderate/high CRF* | | 1.07 (1.03-1.11) | 0.92 (0.85-0.99) |
| **Kidney, low CRF** | 0.09 | Ref | 2.00 (1.61-2.49) |
| *Moderate/high CRF* | | 0.91 (0.82-1.01) | 1.42 (1.18-1.71) |
| **Bladder, low CRF** | 0.01 | Ref | 1.37 (1.08-1.73) |
| *Moderate/high CRF* | | 0.96 (0.88-1.05) | 0.88 (0.72-1.07) |
| **HEMATOLOGICAL CANCERS** | | | |
| **Leukemia, low CRF** | 0.78 | Ref | 1.35 (1.06-1.71) |
| *Moderate/high CRF* | | 1.09 (0.98-1.21) | 1.53 (1.28-1.83) |
| **Myeloma, low CRF** | 0.21 | Ref | 1.43 (0.97-2.10) |
| *Moderate/high CRF* | | 1.25 (1.08-1.46) | 1.33 (1.00-1.76) |
| **Hodgkin's lymphoma, low CRF** | 0.11 | Ref | 1.72 (1.28-2.30) |
| *Moderate/high CRF* | | 1.02 (0.87-1.21) | 1.27 (0.95-1.70) |
| **Non-Hodgkin's lymphoma, low CRF** | 0.007 | Ref | 1.53 (1.26-1.86) |
| *Moderate/high CRF* | | 1.13 (1.03-1.24) | 1.22 (1.02-11.45) |

BMJ Publishing Group Limited (BMJ) disclaims all liability and responsibility arising from any reliance placed on this supplemental material which has been supplied by the author(s)


Cox regression models including an interaction term between dichotomized CRF (low vs. moderate/high) and dichotomized BMI category (underweight/normal weight vs. overweight/obesity), with likelihood ratio testing of the interaction. Adjusted for site, year, and age at conscription as well as parental level of education.
CRF=cardiorespiratory fitness. BMI=Body mass index. HR=Hazard ratio

Supplementary Table 5: Cardiorespiratory fitness at conscription and incidence of cancer (n = 1,077,042)

| Cancer site | n cases | Cardiorespiratory fitness (ref = low) Moderate HR (95% CI) | High HR (95% CI) | *p-value for linear trend*[a] | *P for cognitive ability* |
|---|---|---|---|---|---|
| **Any cancer site** | 64,541 | 1.01 (0.99-1.03) | 1.05 (1.03-1.07) | <0.001 | <0.001 |
| **Malignant skin** | 23,045 | 1.10 (1.07-1.14) | 1.25 (1.21-1.30) | <0.001 | <0.001 |
| **Bronchi and lung** | 1,634 | 0.84 (0.75-0.95) | 0.63 (0.55-0.72) | <0.001 | <0.001 |
| **Head & Neck** | 2,734 | 0.88 (0.80-0.96) | 0.84 (0.76-0.92) | <0.001 | 0.001 |
| **Central nervous system** | 2,012 | 0.94 (0.84-1.04) | 1.00 (0.89-1.13) | 0.85 | 0.60 |
| **Thyroid gland** | 683 | 1.01 (0.84-1.21) | 1.01 (0.82-1.23) | 0.86 | 0.57 |
| **GASTROINTESTINAL CANCERS** | | | | | |
| **Esophagus** | 688 | 0.79 (0.66-0.94) | 0.68 (0.56-0.83) | <0.001 | <0.001 |
| **Stomach** | 902 | 0.89 (0.76-1.04) | 0.83 (0.69-0.98) | <0.001 | 0.002 |
| **Pancreas** | 1,278 | 0.93 (0.82-1.07) | 0.92 (0.79-1.06) | 0.001 | 0.003 |
| **Liver, bile ducts and gallbladder** | 1,109 | 0.86 (0.75-0.99) | 0.66 (0.56-0.77) | <0.001 | <0.001 |
| **Colon** | 3,220 | 0.95 (0.87-1.03) | 0.83 (0.76-0.91) | <0.001 | 0.07 |
| **Rectum** | 2,335 | 1.03 (0.93-1.13) | 0.97 (0.87-1.08) | 0.09 | 0.02 |
| **UROLOGICAL CANCERS** | | | | | |
| **Prostate** | 14,214 | 1.03 (0.99-1.07) | 1.04 (1.00-1.09) | <0.001 | <0.001 |
| **Kidney** | 1,752 | 0.94 (0.74-1.05) | 0.84 (0.74-0.95) | <0.001 | <0.001 |
| **Bladder** | 2,259 | 0.94 (0.85-1.04) | 0.93 (0.83-1.03) | 0.08 | 0.001 |
| **HEMATOLOGICAL CANCERS** | | | | | |
| **Leukemia** | 1,987 | 1.05 (0.94-1.17) | 1.14 (1.02-1.29) | 0.18 | 0.99 |
| **Myeloma** | 915 | 1.15 (0.98-1.35) | 1.21 (1.02-1.44) | 0.03 | 0.96 |
| **Hodgkin's lymphoma** | 842 | 0.95 (0.80-1.12) | 1.02 (0.85-1.23) | 0.74 | 0.33 |
| **Non-Hodgkin's lymphoma** | 2,556 | 1.07 (0.97-1.17) | 1.12 (1.01-1.24) | 0.03 | 0.05 |

Adjusted for year, site, age, body mass index, parental education at conscription, and cognitive ability at conscription where indicated. [a]Linear trends tested utilizing 9-level cardiorespiratory fitness. HR=Hazard ratio

Supplementary table 6. Baseline demographics at conscription for the full target population and the analytic sample.

| | **Sample with information on parental education (N=1,078,000)** | **Study sample (N=1,226,478)** | **Full target population (N=1,811,024** |
|---|---|---|---|
| **Year for conscription, mean (SD)** | 1985 (9) | 1984 (9) | 1987 (10) |
| **Age at conscription, years, mean (SD)** | 18.3 (0.6) | 18.3 (0.7) | 18.3 (0.7) |
| **Years of follow-up, mean (SD)** | 32.2 (9.7) | 32.8 (10.3) | 30.1 (11) |
| **Height, cm, mean (SD)** | 179 (7) | 179 (7) | 179 (7) |
| Missing, n (%) | 0 | 0 | 145,576 (8%) |
| **Body Mass Index (BMI), kg/m$^2$, mean (SD)** | 21.8 (2.8) | 21.7 (2.8) | 21.9 (3.1) |
| Missing | 0 | 0 | 145,800 (8%) |
| **BMI-category** | | | |
| Underweight | 77,361 (7 %) | 92,267 (8 %) | 129,584 (8%) |
| Normal weight | 882,598 (82 %) | 1,002,221 (82 %) | 1,327,781 (80%) |
| Overweight | 101,383 (9 %) | 113,253 (9 %) | 169,551 (10%) |
| Obesity | 16,658 (2 %) | 18,737 (2 %) | 38,308 (2%) |
| Missing | 0 | 0 | 145,800 (8%) |
| **CRF category** | | | |
| Low | 308,974 (29 %) | 365,874 (30 %) | 367,544 (30%) |
| Moderate | 468,677 (43 %) | 519,652 (42 %) | 520,899 (42%) |
| High | 300,349 (28 %) | 340,952 (28 %) | 341,488 (28%) |
| Missing | 0 | 0 | 581,093 (32%) |
| **Muscle strength category** | | | |
| Low | 101,444 (10 %) | 120,089 (10 %) | 182,617 (12%) |
| Moderate | 582,047 (58 %) | 673,495 (58 %) | 857,958 (58%) |
| High | 322,420 (32 %) | 357,933 (31 %) | 451,080 (30%) |
| Missing | 72,089 (7 %) | 74,961 (6 %) | 319,369 (18%) |
| **Systolic blood pressure, mean (SD)** | 128 (11) | 128 (11) | 129 (11) |
| Missing | 3,361 (0.3 %) | 3,439 (0.3 %) | 226,701 (13%) |
| **Diastolic blood pressure, mean (SD)** | 67 (10) | 67 (10) | 68 (10) |
| Missing | 4,298 (0.4 %) | 4,414 (0.4 %) | 227,988 (13%) |
| **Diabetes mellitus** | 381 (0.04 %) | 453 (0.04 %) | 4,605 (0.25%) |
| **Hypertension** | 1,343 (0.12 %) | 1,586 (0.13 %) | 2,661 (0.15 %) |
| **Cardiovascular disease** | 20,769 (1.9 %) | 23,901 (2.0 %) | 41,417 (2.3%) |
| **Kidney disease** | 921 (0.09 %) | 1,062 (0.09 %) | 1,984 (0.11%) |
| **Alcohol abuse** | 1,510 (0.14 %) | 2,300 (0.19 %) | 3,517 (0.19%) |
| **Substance abuse** | 2,258 (0.21 %) | 3,329 (0.27 %) | 5,756 (0.32%) |
| **Parental education** | | | |
| **Compulsory school** | 299,188 (28 %) | 299,188 (28 %) | 397,934 (24%) |
| **High school-≤2 years university** | 602,461 (56 %) | 602,461 (56%) | 942,996 (58%) |
| **>2 years university** | 176,351 (16 %) | 176,351 (16 %) | 287,345 (18%) |
| Missing | 0 | 148,478 (12 %) | 182,749 (10%) |

Supplementary table 7. Sensitivity analysis in population excluding all individuals with preexisting diabetes, hypertension, cardiovascular disease, kidney disease, alcohol abuse, or substance abuse at baseline (n = 1,052,447)

| | | Cardiorespiratory fitness (ref = low) | | |
|---|---|---|---|---|
| **Cancer site** | **n cases** | **Moderate** | **High** | ***p-value for linear trend[a]*** |
| | | **HR (95% CI)** | **HR (95% CI)** | |
| **Any cancer site** | 62,692 | 1.02 (1.00-1.04) | 1.06 (1.04-1.08) | <0.001 |
| **Malignant skin** | 22,434 | 1.13 (1.09-1.16) | 1.31 (1.26-1.35) | <0.001 |
| **Bronchi and lung** | 1,578 | 0.81 (0.72-0.91) | 0.58 (0.51-0.67) | <0.001 |
| **Head & Neck** | 2,658 | 0.86 (0.79-0.95) | 0.81 (0.74-0.90) | <0.001 |
| **Central nervous system** | 1,958 | 0.93 (0.84-1.04) | 0.99 (0.88-1.12) | 0.95 |
| **Thyroid gland** | 664 | 1.02 (0.84-1.23) | 1.03 (0.84-1.26) | 0.97 |
| **GASTROINTESTINAL CANCERS** | | | | |
| **Esophagus** | 664 | 0.76 (0.64-0.91) | 0.62 (0.50-0.75) | <0.001 |
| **Stomach** | 862 | 0.88 (0.75-1.03) | 0.80 (0.67-0.95) | <0.001 |
| **Pancreas** | 1,228 | 0.93 (0.81-1.06) | 0.87 (0.76-1.01) | <0.001 |
| **Liver, bile ducts and gallbladder** | 1,012 | 0.86 (0.74-0.99) | 0.63 (0.53-0.74) | <0.001 |
| **Colon** | 3,136 | 0.94 (0.87-1.02) | 0.83 (0.75-0.91) | <0.001 |
| **Rectum** | 2,275 | 1.02 (0.92-1.12) | 0.94 (0.84-1.04) | 0.02 |
| **UROLOGICAL CANCERS** | | | | |
| **Prostate** | 13,835 | 1.05 (1.01-1.09) | 1.07 (1.03-1.12) | <0.001 |
| **Kidney** | 1,700 | 0.92 (0.82-1.02) | 0.79 (0.70-0.90) | <0.001 |
| **Bladder** | 2,192 | 0.94 (0.85-1.04) | 0.91 (0.82-1.02) | 0.04 |
| **HEMATOLOGICAL CANCERS** | | | | |
| **Leukemia** | 1,930 | 1.05 (0.94-1.17) | 1.15 (1.02-1.29) | 0.11 |
| **Myeloma** | 880 | 1.16 (0.98-1.37) | 1.26 (1.06-1.49) | 0.01 |
| **Hodgkin's lymphoma** | 817 | 0.96 (0.81-1.13) | 1.01 (0.84-1.22) | 0.68 |
| **Non-Hodgkin's lymphoma** | 2,481 | 1.06 (0.96-1.17) | 1.12 (1.01-1.24) | 0.03 |

Adjusted for year, site, age, body mass index, and parental education at conscription. [a]Linear trends tested utilizing 9-level CRF. CRF=cardiorespiratory fitness. HR=Hazard ratio

Supplementary table 8. Analyses in the full study sample, not adjusted for parental level of education (n = 1,226,478)

| | | **Cardiorespiratory fitness (ref = low)** | | |
|---|---|---|---|---|
| **Cancer site** | **n cases** | **Moderate** | **High** | ***p-value for linear trend***[a] |
| | | **HR (95% CI)** | **HR (95% CI)** | |
| **Any cancer site** | 84,117 | 1.03 (1.01-1.04) | 1.06 (1.04-1.08) | <0.001 |
| **Malignant skin** | 28,290 | 1.14 (1.10-1.17) | 1.34 (1.30-1.38) | <0.001 |
| **Bronchi and lung** | 2,567 | 0.79 (0.72-0.87) | 0.58 (0.52-0.64) | <0.001 |
| **Head & Neck** | 3,544 | 0.88 (0.81-0.95) | 0.82 (0.75-0.89) | <0.001 |
| **Central nervous system** | 2,840 | 0.96 (0.88-1.05) | 1.03 (0.94-1.14) | 0.24 |
| **Thyroid gland** | 822 | 1.02 (0.87-1.21) | 1.01 (0.84-1.21) | 0.85 |
| **GASTROINTESTINAL CANCERS** | | | | |
| **Esophagus** | 1,009 | 0.77 (0.67-0.89) | 0.63 (0.54-0.74) | <0.001 |
| **Stomach** | 1,292 | 0.90 (0.79-1.02) | 0.77 (0.67-0.89) | <0.001 |
| **Pancreas** | 1,859 | 0.97 (0.87-1.08) | 0.86 (0.77-0.97) | <0.001 |
| **Liver, bile ducts and gallbladder** | 1,593 | 0.79 (0.71-0.89) | 0.56 (0.49-0.65) | <0.001 |
| **Colon** | 4,301 | 0.96 (0.90-1.03) | 0.81 (0.75-0.88) | <0.001 |
| **Rectum** | 3,137 | 1.01 (0.93-1.10) | 0.91 (0.83-1.00) | 0.002 |
| **UROLOGICAL CANCERS** | | | | |
| **Prostate** | 19,679 | 1.07 (1.03-1.11) | 1.11 (1.07-1.15) | <0.001 |
| **Kidney** | 2,294 | 0.91 (0.83-1.00) | 0.76 (0.69-0.85) | <0.001 |
| **Bladder** | 3,070 | 0.94 (0.87-1.03) | 0.88 (0.80-0.96) | <0.001 |
| **HEMATOLOGICAL CANCERS** | | | | |
| **Leukemia** | 2,730 | 1.05 (0.96-1.15) | 1.11 (1.00-1.22) | 0.17 |
| **Myeloma** | 1,213 | 1.13 (0.98-1.30) | 1.21 (1.05-1.40) | 0.004 |
| **Hodgkin's lymphoma** | 1,038 | 1.00 (0.86-1.16) | 1.11 (0.94-1.30) | 0.48 |
| **Non-Hodgkin's lymphoma** | 3,250 | 1.06 (0.97-1.15) | 1.09 (1.00-1.20) | 0.06 |

Adjusted for year, site, age, and body mass index at conscription. [a]Linear trends tested utilizing 9-level CRF. CRF=cardiorespiratory fitness. HR=Hazard ratio
